# Artificial Intelligence and the future of radiotherapy planning: The Australian radiation therapists prepare to be ready

**DOI:** 10.1002/jmrs.791

**Published:** 2024-04-20

**Authors:** Vanessa Panettieri, Giovanna Gagliardi

**Affiliations:** ^1^ Department of Physical Sciences Peter MacCallum Cancer Centre Melbourne Victoria Australia; ^2^ Sir Peter MacCallum Department of Oncology The University of Melbourne Melbourne Victoria Australia; ^3^ Central Clinical School Monash University Melbourne Victoria Australia; ^4^ Department of Medical Imaging and Radiation Sciences Monash University Clayton Victoria Australia; ^5^ Medical Radiation Physics Department Karolinska University Hospital Stockholm Sweden; ^6^ Department of Oncology‐Pathology Karolinska Institutet Stockholm Sweden

## Abstract

The use of artificial intelligence (AI) solutions is rapidly changing the way radiation therapy tasks, traditionally relying on human skills, are approached by enabling fast automation. This evolution represents a paradigm shift in all aspects of the profession, particularly for treatment planning applications, opening up opportunities but also causing concerns for the future of the multidisciplinary team. In Australia, radiation therapists (RTs), largely responsible for both treatment planning and delivery, are discussing the impact of the introduction of AI and the potential developments in the future of their role. As medical physicists, who are part of the multidisciplinary team, in this editorial we reflect on the considerations of RTs, and on the implications of this transition to AI.

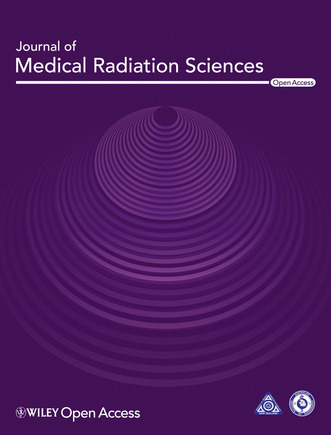

In the fast‐evolving field of radiation oncology, complex solutions are now being developed in order to transition away from a standardised homogenous irradiation approach towards more complex deliveries.^1^ This evolution has no doubt been facilitated by the introduction of advanced treatment planning and delivery methodologies based on modulated approaches (such as volumetric modulated arc therapy and intensity modulated radiation therapy). These techniques, allowing the sculpting of doses around organs at risk while also maintaining the required tumour coverage using complex optimisation algorithms, are now commonly used worldwide. In parallel, planning quality has been linked in numerous studies to patient outcomes.^2^, ^3^ For some treatment areas, outcomes are also correlated with anatomical variations during delivery that could benefit from a daily adaptation of the treatment.^4^ Examples of these variations are widely reported in the literature and range from short‐term anatomical changes such as those due to respiratory motion or pre‐treatment preparation (i.e. bowel or bladder filling for pelvic treatments) or longer ones due to tumour regression or weight loss (i.e. head and neck treatment).^5^ All of these processes, which have traditionally been heavily reliant on human skills, are time‐consuming and based on the operator's experience. Therefore, developments have been in progress for a number of years to improve automation to allow the rapid adaptation and standardisation of treatment delivery.

In the last decade, we have seen the introduction of automation by means of machine learning algorithms. These algorithms, which are based on processes used by artificial intelligence (AI) systems to imitate intelligent human behaviour,^6^ can learn from high‐quality plans or exploit automated rule implementation,^7^ to generate predictions that can also be shared among institutions promoting harmonisation.^8^ While this change in the field of radiation oncology might seem to be disruptive, this ‘supervised’ automation has evolved alongside more traditional approaches for a number of years, using current outcomes and expertise to prepare training datasets in model building. There seems to be, however, the potential for the development of ‘intelligent’ processes, suggesting, in other words, that we have only been ‘scratching the surface’ of the potential of AI‐driven automation.^6^ In the same way, the use of AI has been assimilated into many aspects of society, which has adapted to it as in an evolutionary process.^9^ This use of AI has accelerated in the last decade due to renewed interest in the use of ‘deeper’ neural networks which are inspired by the complex design of neural connections in the human brain. These neural networks could potentially surpass humans in the ability to learn and address complex problems in a fraction of the time,^6^ suggesting a paradigm shift also in radiation therapy on how some tasks might be performed in the future, as an example in the overall process of treatment planning (TP).

## What if treatment planning is fully AI based in 5 to 10 years?

While TP has traditionally been approached using the same principles, that is manually separating contouring, plan creation, dose volume histogram (DVH)‐based optimisation and dose calculation, AI can already facilitate a fully automated process in which a plan is now built as an image reconstruction problem. This approach has the potential to directly predict three‐dimensional (3D) dose information given an anatomical input in a matter of just minutes,^6^, ^10^, ^11^, ^12^ eliminating the need for human interaction in the generation phase. AI will expand the ability to rapidly create a personalised treatment for each patient and at each stage of their treatment journey. As clearly explained by Jones et al.,^13^ in their recently published commentary, this evolution represents not only an opportunity, but also cause for some concern both for the evolution of the current processes and the future of the multidisciplinary team (MDT). In particular, in countries like Australia where the radiation therapists (RTs) are largely responsible for both treatment planning and delivery, discussions are in progress in preparation for safely transitioning to automation with AI. In Jones et al.,^13^ table 1 summarises some key points considered crucial for this transition that include, among others, the establishment of a multi‐professional core group, which will include RTs, medical physicists (MP), radiation oncologists (ROs) but will also rely on the expertise of other professions (such as IT specialists and radiation engineers), that can be engaged in the deployment of these tools. This is paramount to ensure that the same high‐level quality assurance and control, as currently used in human‐based processes, can be developed in AI‐driven ones keeping in mind that the ethics and standards implications, ranging from data sourcing reliability to build the algorithms to the need of AI‐dedicated regulation and governance, are still not resolved.^13^, ^14^ These considerations might also need adjustment while we harness the power of this continuously evolving technology. AI has the potential to dictate treatment choices from diagnosis to plan preparation, and all the way to treatment evaluation, by the incorporation of all of the patients' related data in an automated way. Jones et al.^13^ point out that this shift is bound to have a large impact also on the roles and responsibilities of the RTs, along with the whole MD team. We envision that the MDT will have to re‐think or re‐develop some of the current tasks, and even relationships, among the different craft groups to adapt to the changes introduced by AI tools. Some processes that will be impacted by automation (such as time‐consuming patient‐specific routine QA for MP) will naturally require less staffing, allowing MDT members that are performing more repetitive tasks to be redeployed to other roles, or further up‐skilling to deal with more challenging tasks requiring human input. The Radiation Oncology workforce seems bound to change to become less task‐driven and more thought‐driven, opening up new collaborative opportunities within the MDT. In particular, for the RTs planning, preparation and checking times might be reduced, potentially reducing headcounts for these tasks. While this change could cause anxiety for the future of the profession, the authors highlight a survey^15^ that suggests RTs are generally optimistic that, with adequate preparation, these developments will allow more focus on patient process individualisation in the whole treatment chain (for instance in treatment adaptation). This in turn will allow more time to collaborate with RO and MP in decision‐making and clinical reasoning, in particular, for complex cases. This prospect might also enhance research opportunities focusing on large‐scale evaluations across many aspects of the radiation therapy process, both in clinical trials, and real‐world scenarios. Especially in this developmental phase, AI methods will still learn from expert practitioners, meaning RTs will still be the driving force when high‐quality data needs to be produced and evaluated for model building. As part of the MDT, RTs with time will contribute to the understanding of model outputs and failures (interpretability), monitoring their performance and ensuring that these models are generalisable.

So, the future is full of possibilities; however, it is important to be prepared. Education will be essential, both in academic and clinical settings, for practitioners to ensure adequate competence with these new developments, currently perceived mostly as a ‘black box’.^6^ An important point discussed by Jones et al.^13^ is maintaining the expertise and skills that allow a critical oversight when these new automated tools are introduced to the clinic. It will be a balancing act, however, to make sure that a more traditional knowledge of planning tasks is still transmitted to new staff in the workforce while also promoting the understanding and the safe use of AI advanced tools. Training and professional development will have to incorporate dedicated time to develop both skills, aided by the fact that the transition between the two in the next years will not be a binary process but more of a side‐by‐side evolution.

Importantly, while it is the duty of the whole MDT to be ready for this exciting future, we cannot forget the importance of patient experience and their involvement in the transition to AI. As highlighted by Jones et al.,^13^ patients surveyed in the UK have expressed moderately negative views towards having AI drive their treatment. It is therefore our role to reflect on this, establish a dialogue and collaborate with patients to shape AI into a tool that is not meant to replace humans and human skills, but rather, to enhance them. In parallel to these technologies becoming part of our day‐to‐day clinical routine, clear and transparent communication strategies will also have to be developed during this transition to avoid distrust in AI methods and results. In the currently discussed scenarios, AI‐based interventions might also have the potential to benefit areas of the world where access to radiotherapy is still limited by workforce and equipment shortages, so it is yet and again important to proceed with caution and awareness.

Finally, it should also be kept in mind that while efficiency, standardisation and accuracy are bound to increase with automation, questions have arisen regarding how overuse of AI may hinder innovation through human creativity. Relying too much on AI could impact opportunities which in the past, have led to paradigm shifts in radiation oncology (e.g. the introduction of stereotactic treatments^16^, ^17^). However, it is too early to say. At this stage it is not clear in which direction these developments are going, but, it is plausible that, in the context of the radiotherapy chain, AI‐based approaches might promote and assist innovation by reducing those repetitive tasks that lend themselves to automation. These methods will allow gains in efficiency and will have the potential to free and adequately prepare human resources in the MDT. These resources can instead focus creatively on treatment development and quality as a whole, ultimately improving personalised care and treatment outcome, as shown by the example of the preparative discussions of the Australian RTs.

## Conflict of Interest

The authors declare no conflict of interest.

## Data Availability

Data sharing not applicable to this article as no datasets were generated or analysed during the current study.
